# Negative Priming in a Joint Selection Task

**DOI:** 10.1371/journal.pone.0042963

**Published:** 2012-08-09

**Authors:** Timothy N. Welsh, Laura M. McDougall

**Affiliations:** 1 Faculty of Kinesiology and Physical Education, University of Toronto, Toronto, Ontario, Canada; 2 Faculty of Kinesiology, University of Calgary, Calgary, Alberta, Canada; Centre Hospitalier Le Vinatier (Bât. 452), France

## Abstract

Recent studies have suggested that the observation of another individual executing a movement activates representations of the observed movement in the observer. These representations are thought to be used by other systems to facilitate a variety of social cognitive processes, such as social searches. Previous research on social searches has primarily involved contexts where targets were presented in isolation. Typical environments, however, contain targets and non-targets and one must select the correct information for task completion. To gain insight into the processes underlying social searches, participants completed negative priming tasks alone and in pairs. Results indicated that there were no differences in the negative priming effects resulting from the participants observed or performed the preceding selection task. Further, the correlations between individual and joint negative priming suggest that similar processes were activated on these tasks. The findings support the co-representation hypothesis and provide insight into the processes underlying selection in individual and social settings.

## Introduction

Humans are social animals who are constantly observing the behaviors of others to achieve a variety of intra- and inter-individual goals. The results of the research into the processes underlying action observation suggest that the observation of an action activates a sensorimotor representation of that response in the observer. It is thought that this observation-evoked response code is then accessed by other cognitive systems for a variety of purposes including communication [1], learning [2], joint action [3], and the understanding of other people’s mental states [4]. Hence, it is thought that this action co-representation allows individuals to understand what other people are doing, to predict what they might do next, and/or to coordinate actions with them to achieve common goals.

Much of the support for the hypothesis that the observation or knowledge of another person’s response activates response codes in the observer is derived from studies of joint action tasks (e.g., [5], [6]; see [7] for a review). The typical joint action study involves participants completing a task, such as a spatial compatibility task, alone and with a partner (e.g., [8]). In the latter joint task condition, the whole task is divided between the partners such that each individual is only responsible for completing a part of the task. Hence, in these joint tasks, each individual is working relatively independently, but together with the partner the whole task is completed. The key finding of these studies is that the behavioral effects observed when individuals complete the whole task on their own are also observed when pairs of individuals perform the task together, even though each individual in the pair is only responsible for completing a portion of the task. It has been suggested that these joint action effects occur because each individual in the pair represents the actions of the partner and that these co-represented response codes subsequently engage the same series of processes that are activated when the individual completes the whole task alone [5]–[9]. In support of the notion that similar sets of processes are activated on individual and joint tasks, we [9] recently found a significant positive correlation between the magnitude of the inhibition of return effect in an individual task and the magnitude of the effect in a joint task. That is, even though there was between-subjects (i.e., individual) variability in the magnitude of effects across both tasks, magnitudes of the effects for a given individual were similar across the different tasks (see also [10]–[11]). Overall, the extant data from joint action studies suggest that, despite the fact that each individual is only responsible for a part of the task, it is as though they are performing the whole task.

Thus far, the research on action observation and joint action has mainly involved contexts in which target information is presented in isolation. As such, the current state of knowledge is that observers represent another person’s target response. It is, however, rare that we perform actions in simple environments in which targets are presented in isolation. We are more often faced with complex situations in which we must select between target and non-target information. This realization led us to question whether observers only co-represent the target response or if observers co-represent the entire selection process of their partner. From the point of view of social cognition and the achievement of common behavioral goals, observers should not only want to represent and understand what another person has done, but observers should also want to understand what another person has selected against because the selection would provide information about what is undesirable.

The present study was designed to determine the extent of co-representation by investigating if observers demonstrate a spatial negative priming (NP) effect following the action of another individual. Spatial NP is the phenomenon of longer response times (RTs) for targets on trial “n” (probe display) when the target is presented in the same location as the distractor on trial “n−1” (prime display) [12]. The dominant explanation of NP is the inhibition hypothesis [12]–[15]. According to this account, the cognitive system, as part of the selection process, actively inhibits and suppresses the distractor information and response on the first (prime) display. If the second (probe) target is then presented in the same location as the distractor in the prime display, the system must overcome residual inhibition associated with the previous selection to activate the previously inhibited response. Overcoming the residual inhibition requires time and effort and leads to increased RTs on these trials relative to controls trials in which the target is presented at other locations.

The main purpose of the present study was to determine if NP is present in the behavior of an observer after the observer has witnessed someone else complete a selection task. Consistent with previous research on NP (e.g., [12]), the study consisted of three probe conditions. In the target repetition condition (TR), the target on the probe display was presented in the same location as the target on the prime display. In the ignored repetition (IR) condition, the probe target was presented at the same location as the distractor in the prime display. Finally, in the control condition, the probe target and distractor were presented in different locations from those in the prime display. The control condition was the baseline from which the influence of the previous target and distractor (i.e., NP) was measured.

Participants in the present study completed the NP task on their own (they were responsible for responding on both prime and probe displays) and with a partner (one participant responded on the prime display and the other participant responded on the probe display). It was predicted that if observers represent the entire selection process of their partner, then the observer’s RTs will be longer when their targets are presented at the same location as their partner’s distractor than when the target is presented in a new location – a joint NP effect. As a further testing of the hypothesis of co-representation and of similar processes on individual and joint tasks, the magnitudes of the NP effects on individual and joint tasks were correlated. Based on previous work [9], [10], it was predicted that, if similar processes underlie performance in the two conditions, then there should be a significant positive correlation between the magnitudes of the NP effects in the two tasks. Alternatively, if observers do not represent the selection process, then a joint NP effect will not be observed and/or there should not be a significant correlation between the magnitudes of the NP effects in the two conditions.

## Methods

### Ethics Statement

All procedures were approved by the University of Calgary Research Ethics Board and complied with the ethical standards of the 1964 Declaration of Helsinki regarding the treatment of human participants in research. All participants provided written informed consent prior to beginning the experiment.

### Participants

Seven pairs of people (7 men, 7 women) from the University of Calgary community completed the experiment. Participants were right-handed, aged 19 to 32 years, were naïve to the purpose of the study, had normal or corrected-to-normal vision, and were financially compensated.

### Apparatus, Stimuli, and Task

**Figure 1:**
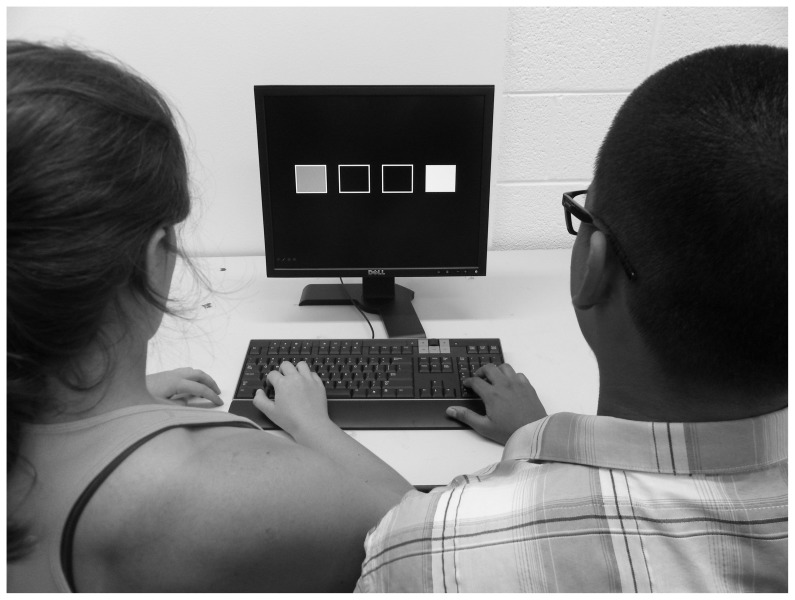
Picture of the apparatus and set-up for the joint protocol. The same arrangement was used for the individual condition except that only one participant was present.

Participants sat beside each other in chairs that were arranged in front of a table. On the table, there were a standard (QWERTY) English keyboard and a 19-inch flat-screen ViewSonic LCD monitor (Figure 1). Participant 1 sat on the left side of the screen and was instructed to respond using the keys “z”, “x”, “c” and “v”. Participant 2 sat on the right side of the screen and was instructed to respond using the keys “1, “2”, “3” and “ENTER” on the number pad. The participants kept the four fingers of the right hand over each of the buttons throughout a block of trials.

**Figure 2:**
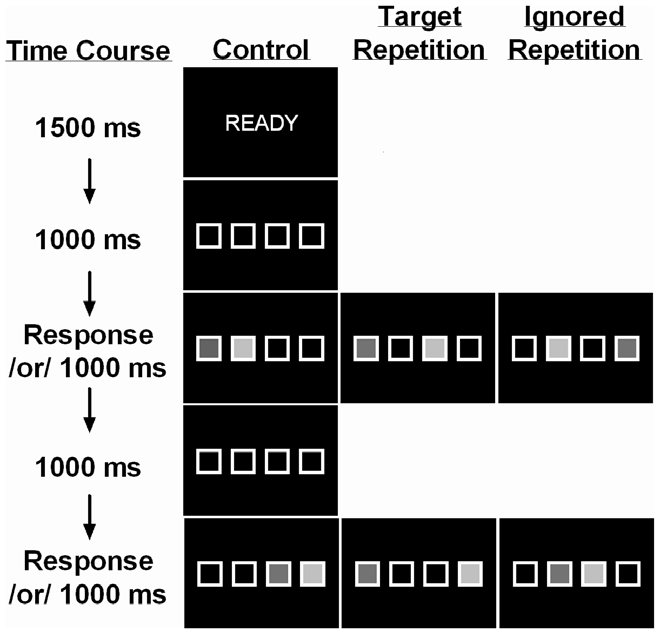
Time course of displays during each trial type. The darker grey squares represent targets and the lighter grey squares represent distractors.

Five display screens appeared in every trial (Figure 2). The first two screens were a “Ready!” warning for 1500 ms followed by the appearance of the four empty placeholders for 1000 ms. The four potential target locations were 60×60 mm squares (1 mm white borders) arranged in a row across the center of black background. Placeholders were separated by 30 mm. The next screen was the first of two response screens - the prime display. After the participant responded to the prime display, the placeholders became empty for another 1000 ms until the second response screen appeared – the probe display. Prime and probe displays remained visible until the participant responded or 1000 ms elapsed. After the response to the probe display was recorded, the screen went blank for 1500 ms at which point the “Ready!” warning screen reappeared informing participants that the next trial was beginning.

Each prime and probe display contained two stimuli - a target and a distractor. The target was a green square that filled one of the placeholders and the distractor was a red square that filled another of the placeholders. Participants were told that the location of the target and distractor were in a random on each of the display screens. The task of the participant was to press the spatially compatible key as soon as they identified the location of the target (a choice localization task). Participant 1 pressed the “z”, “x”, “c”, or “v” key with the index, middle, ring, or pinky finger when the target was in the far left, middle left, middle right, or far right position, respectively. Likewise, Participant 2 pressed the “1”, “2”, “3” or “ENTER” key with the index, middle, ring, or pinky finger when the target was in the far left, middle left, middle right, or far right position, respectively. Participants were told to ignore the distractor that was presented on each display. Custom software created using E-Prime (v1.1) controlled the presentation of the stimuli and recorded the time and identity of each response.

### Experimental Design

Each person of each pair completed the protocol three times: once as an individual in which the participant was to respond on both prime and probe displays, and twice in a joint condition in which they completed the task with the other participant (once with Participant 1 responding to the prime and Participant 2 responding to the probe and once with the order reversed). The order in which the pairs completed the protocols was randomized.

Two blocks of 72 prime/probe display pairs were completed in each protocol. The 72 trials in each of the blocks consisted of 24 control trials (prime and probe targets and distractors were presented in different locations), 24 IR trials (probe target presented at the same location as the prime distractor), and 24 TR trials (probe target presented at the same location as the prime target) presented in a random order (see Figure 2). Probe distractors were always presented in a previously unoccupied location. Targets and distractors were presented an equal number of times in each placeholder in each prime-probe pairing. Overall, each participant completed a total of 432 trials and there were 48 trials for each of the conditions in each of the tasks.

## Results

RT (time elapsed from stimulus onset until key press) and response identity were recorded with each key press. Only probe RTs were analyzed. RTs shorter than 100 ms or longer than 1000 ms were removed (approximately 1% of the trials). RTs for trials on which an incorrect key was pressed were made in either the probe or the preceding prime display trials were also removed (approximately 7% of the data). Once the error data were removed, mean RTs were calculated and initially submitted to a 2 (Task: individual, joint) ×3 (Target: control, TR, IR) within-subjects ANOVA. Alpha was set at 0.05 for all tests.

**Figure 3:**
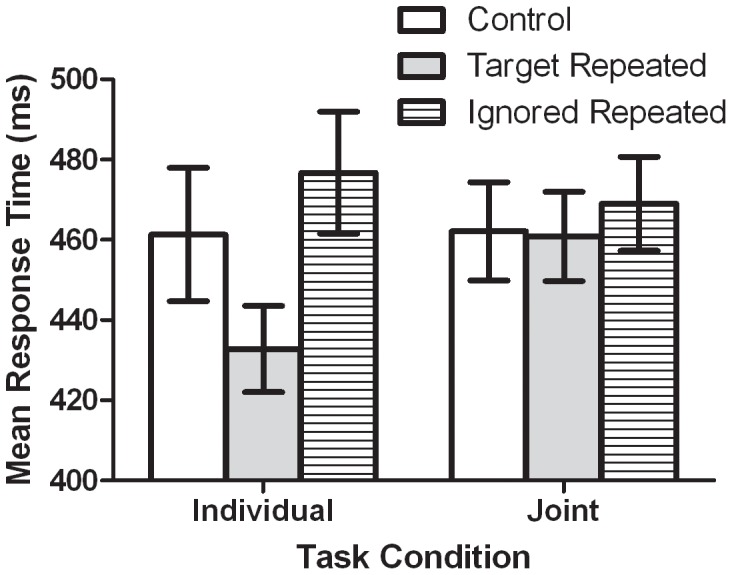
Mean response times (ms) as a function of Person and Target. Open white bars represent values for control trials. Light grey bars represent values for target repetition trials. Bars with horizontal stripes represent values for ignored repetition trials. Standard error of the mean bars are shown.

The ANOVA for RTs revealed a significant main effect of Target, *F*(2, 26) = 16.29, *p*<0.001, and a significant interaction between Task and Target, *F*(2, 26) = 14.79, *p*<0.001. As can be observed in Figure 3, the main value driving the interaction was the short RTs in the individual TR condition. As will be readdressed in the General Discussion, the absence of a TR effect in joint protocol suggests that the short RTs on the TR trials in the individual condition was due to a response priming, as opposed to a perceptual priming, effect.

Given that the differences in individual and joint TR effects led to the significant interaction, the RT data for TR trials were removed from the analysis and the data for control and IR trials were submitted to a 2 (Task: individual, joint) ×2 (Target: control, IR) within-subjects ANOVA. The only significant effect for RTs was a main effect for Target, *F*(1, 13) = 6.02, *p<*0.05. Consistent with previous NP studies, RTs on IR trials (473 ms) were longer than those on control trials (462 ms). The absence of a significant interaction between Task and Target indicates that the NP effect in individual and joint trials was not statistically different, *F*(1, 13) = 3.68, *p>*0.05 (Figure 3). The analysis of percent probe keypress errors using the same 2 (Task) by 2 (Target) ANOVA model likewise revealed that there were more errors in the IR trials (8.78%) than on control trials (5.65%), *F*(1, 13) = 6.66, *p<*0.05. There was no interaction between Task and Target, *F*(1, 13)<1 (Table 1). These data suggest that the mechanisms that cause NP were activated following the performance *and* observation of a selection task.

**Table 1:** Mean and standard deviation () of the percentage button press errors on probe trials.

TrialType	Control	TargetRepetition	IgnoredRepetition
Individual	6.25 (4.5)	2.91 (3.0)	10.12 (6.3)
Joint	5.06 (5.46)	4.76 (3.6)	7.44 (5.2)

**Figure 4:**
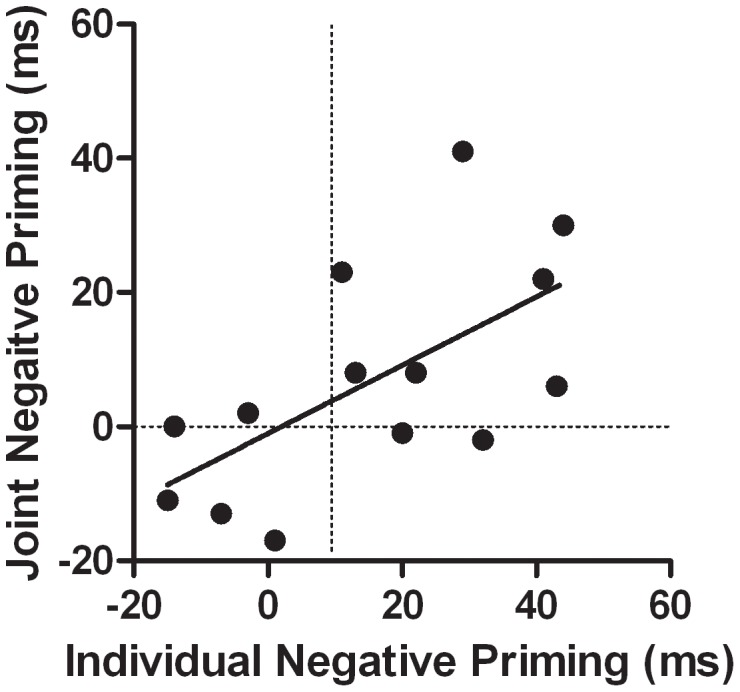
Correlation of the negative priming effects (ms) between the individual and joint conditions.

Consistent with other social cognitive effects, it is suggested that the observer represented the performance of their partner and that the same mechanisms that caused NP on individual trials caused NP on joint trials. In support of the co-representation hypothesis, the magnitude of the NP effects (RTs for IR trials minus RTs for control trials) in both protocols were highly and significantly correlated, *r* = 0.63, *p*<0.05 (Figure 4). Similar correlations between individual and joint information processing effects have recently been observed in other social search and response tasks [9].

## Discussion

The present study investigated the extent to which people represent the task performance of others. It was hypothesized that people represent the entire task of the person they observe and thus would represent both target and non-target information and engage in a selection process. The results of the present studies support this hypothesis because the NP effects following the performance of a selection task were not different from the NP effects following the observation of someone else performing the same selection task. Critically, the magnitudes of these two effects were highly correlated suggesting a similar set of processes causing these effects [9], [10]. The results of these studies contribute to our understanding of social and independent searches in at least two ways.

First, the results of the study reveal an NP effect following the observation of a selection. The key additional finding was that the magnitude of the NP effects in the individual and joint tasks were highly correlated, suggesting that similar inhibitory processes were activated during the observation and performance of the selection task. These data suggest that observers represented the selection of their partners and engaged the same set of inhibitory processes that are activated when the individual actually performs the selection task [9]. These data and conclusions are consistent with and extend a recent study by Frischen et al. [16].

In the study by Frischen et al. [16], participants sat across from an experimenter and completed a series of aiming movements to targets that were presented at one of four locations arranged in a square. The trials were completed in prime-probe pairs and distractors were presented on both prime and probe displays. On some blocks of trials, the participant completed the selection task on both the prime and probe displays and on other blocks the experimenter completed the selection task on the prime display and the participant completed the selection on the probe display. Previous research on NP in selective reaching tasks when individuals are working alone has shown that the NP effects at locations close to the participant are larger than the NP at locations farther from the participant. This pattern of NP has been taken as evidence for the utilization of an action-centered distribution of attention wherein distractors that afford efficient responses receive greater inhibition than those that afford less efficiently completed responses [17]–[18].

The key result of the Frischen et al. [16] study was that the pattern of greater NP at near locations observed when the participants completed both prime and probe responses was reversed when the participant completed the probe response after the experimenter completed the prime response such that greater NP was now observed at the farther locations (i.e., the locations closer to the experimenter). Frischen et al. [16] concluded that this reversal in the pattern of NP occurred because the participant observed and represented the selection and performance of the experimenter from the perspective of the experimenter. The reversed pattern of NP developed because the distractors closer to the experimenter (farther from the participant) afforded more efficient responses for the experimenter than those farther from the experimenter (closer to the participant) and thus received more inhibition. This greater inhibition subsequently caused greater NP for the locations farther from the participant (closer to the experimenter) when the participant responded on the probe trial.

Consistent with the conclusions of Frischen et al. [16], we suggest that the pattern of effects observed here developed because, when an individual observes another person performing a selection task, the observer represents both the target and non-target information and engages the processes of selection as though they were performing the selection. Thus, the observer inhibits the response to the partner’s distractor as though they were performing the selection and this inhibition subsequently affects response programming on the probe trial. Such a complete degree of representation is consistent with the notion that action observation provides us with information about the goals of other people because, through a representation of the selection process, the observer might gain insight into the reasons another individual may select one object over another. In addition, these findings fit with a evolutionary interpretation of the development of the mechanisms activated during the selection process [12], [19] because actively inhibiting what other people consider to be non-target information would increase the efficiency of an individual’s search pattern and allow many people to coordinate search patterns.

The second important finding was that the facilitation effect on TR trials was observed when a response was required on the prime display, but was not observed when a response was not required on the prime display. This pattern of effects suggest that the facilitation effect is due to a motor facilitation rather than a perceptual priming because if perceptual priming associated with color repetition was responsible for the facilitation, then a facilitation should have been observed in all task conditions. This conclusion is also based on previous work showing similar facilitation effects associated with repeated response execution [20]–[21].

To elucidate, in a study that was similar to the present work, Welsh et al. [6] observed a facilitation in movement execution (shorter movement times) for repeated relative to different actions that was only present when the participants actually completed the movement. Participants in the Welsh et al. [6] study worked in pairs and completed aiming movements to targets that appeared in one of two positions. These movements were completed in an alternating-paired pattern (an AABBAABB…) such that participant A made two responses, then participant B made two responses and so on. Despite finding an inhibitory effect in reaction time where reaction times to repeated targets were longer than reaction times to different targets (i.e., an inhibition of return effect [22]–[23]), a facilitation effect associated with repeated movements was observed in movement time. It is important to note that, although the inhibitory effect in reaction time was observed in both individual (e.g., AA) and joint (e.g., AB) trial types, the facilitation in movement time for a repeated movement only occurred on individual trials when participants actually executed the task (e.g., AA trial types). There was no facilitation in movement execution following the observation of movements (e.g., AB trial types). It was argued that the motor facilitation effects only occurred in the individual condition because the response was fully planned and executed and that residual activity associated with a recently executed response was maintained in motor or working memory systems [20]–[21]. This residual activity then decreased the amount of time required to activate that same response when the response has to be reactivated relative to when the movement has to be reprogrammed. Because the response was not fully planned and executed when the actor observed their partner (on joint trials), there was no residual activity from that response and, hence, no facilitation.

A similar residual motor activity/working memory mechanism can account for the pattern of RTs (a sum of reaction and movement time) on the TR trials. That is, the facilitation effects on TR trials in the individual condition may have emerged because the activated and selected target response was fully planned and executed. The residual activity and/or memory trace of the response in the motor system from the execution facilitated response planning and initiation in the subsequent trial. On the other hand, because the target response was not actually executed on joint trials, the response codes did not reach the same level of activation on joint trials, especially in the motor system. Without activation of the response codes in the motor system, there would be no facilitation in the joint condition. This is not to suggest that the motor system was not activated on observation trials because there is substantial evidence that there is motor system activation during observation (e.g., [24]). The point here is that the executed response code reaches a higher degree of activation and that it is the higher degree of activation that results in the facilitation effect in the individual TR trials relative to the joint condition. Further, it is important to note that, according to this account, the selection and activation/execution of the responses are linked, but are relatively independent. For this reason, the observation of the stimuli and the response was sufficient to cause generate the mechanisms of NP in the selection process and modulate future selection, while not being of sufficient magnitude to influence programming and execution (see also [6]). Overall, the positive priming effect on TR trials has not received much attention and would benefit from further investigation.

In sum, the results of the present studies suggest that the completion of joint action tasks involves co-representation of the co-actors performance. In particular, the correlation between the magnitudes of the individual and joint NP effects is consistent with the notion that partners represent the performance of the other individual and that these representations activate the same set of processes that are activated when people perform the task [3], [5], [6], [9]. Although participants in the present study seemed to spontaneously engage in co-representation because there was no direct common goal to the task, it is suggested that this process of response co-representation is a key mechanism facilitating the completion of joint actions with more explicit common goals.
